# Distinct and stage-specific contributions of TET1 and TET2 to stepwise cytosine oxidation in the transition from naive to primed pluripotency

**DOI:** 10.1038/s41598-020-68600-3

**Published:** 2020-07-21

**Authors:** Christopher B. Mulholland, Franziska R. Traube, Enes Ugur, Edris Parsa, Eva-Maria Eckl, Maximilian Schönung, Miha Modic, Michael D. Bartoschek, Paul Stolz, Joel Ryan, Thomas Carell, Heinrich Leonhardt, Sebastian Bultmann

**Affiliations:** 1 Department of Biology II and Center for Integrated Protein Science Munich (CIPSM), Ludwig-Maximilians-Universität München, Planegg-Martinsried, Germany; 2 Department of Chemistry and Center for Integrated Protein Science Munich (CIPSM), Ludwig-Maximilians-Universität München, Munich, Germany; 3 Department of Neuromuscular Disease, UCL Queen Square Institute of Neurology, London, UK

**Keywords:** Cell biology, Embryonic stem cells, Epigenetics, DNA methylation

## Abstract

Cytosine DNA bases can be methylated by DNA methyltransferases and subsequently oxidized by TET proteins. The resulting 5-hydroxymethylcytosine (5hmC), 5-formylcytosine (5fC), and 5-carboxylcytosine (5caC) are considered demethylation intermediates as well as stable epigenetic marks. To dissect the contributions of these cytosine modifying enzymes, we generated combinations of *Tet* knockout (KO) embryonic stem cells (ESCs) and systematically measured protein and DNA modification levels at the transition from naive to primed pluripotency. Whereas the increase of genomic 5-methylcytosine (5mC) levels during exit from pluripotency correlated with an upregulation of the de novo DNA methyltransferases DNMT3A and DNMT3B, the subsequent oxidation steps turned out to be far more complex. The strong increase of oxidized cytosine bases (5hmC, 5fC, and 5caC) was accompanied by a drop in TET2 levels, yet the analysis of KO cells suggested that TET2 is responsible for most 5fC formation. The comparison of modified cytosine and enzyme levels in *Tet* KO cells revealed distinct and differentiation-dependent contributions of TET1 and TET2 to 5hmC and 5fC formation arguing against a processive mechanism of 5mC oxidation. The apparent independent steps of 5hmC and 5fC formation suggest yet to be identified mechanisms regulating TET activity that may constitute another layer of epigenetic regulation.

## Introduction

DNA methylation plays critical roles in the epigenetic regulation of gene expression and genome stability in mammals^1^. During mammalian development, methylated cytosine (5mC) serves as a critical epigenetic barrier to guide cell fate decisions and restrict developmental potential^2^. Genomic 5mC patterns are established by the de novo DNA methyltransferases DNMT3A and DNMT3B and maintained through subsequent cell divisions by DNMT1^3^. The mitotic inheritance of 5mC constitutes a form of epigenetic memory enabling the long term maintenance of cell identity. Extinguishing such memory requires extensive epigenetic reprogramming and is key for the acquisition of naive pluripotency (i.e. the capacity of cells to contribute to all lineages in the embryo) during development^4^. In mammals, genome-wide erasure of 5mC accompanies the restoration of developmental potential following fertilization, reaching a nadir in the naive pluripotent inner cell mass (ICM) of the pre-implantation blastocyst^5–7^. In turn, the transition from a naive pluripotent state to one “primed” for lineage commitment upon implantation coincides with the establishment of global DNA methylation patterns^8–10^.

**Figure 1:**
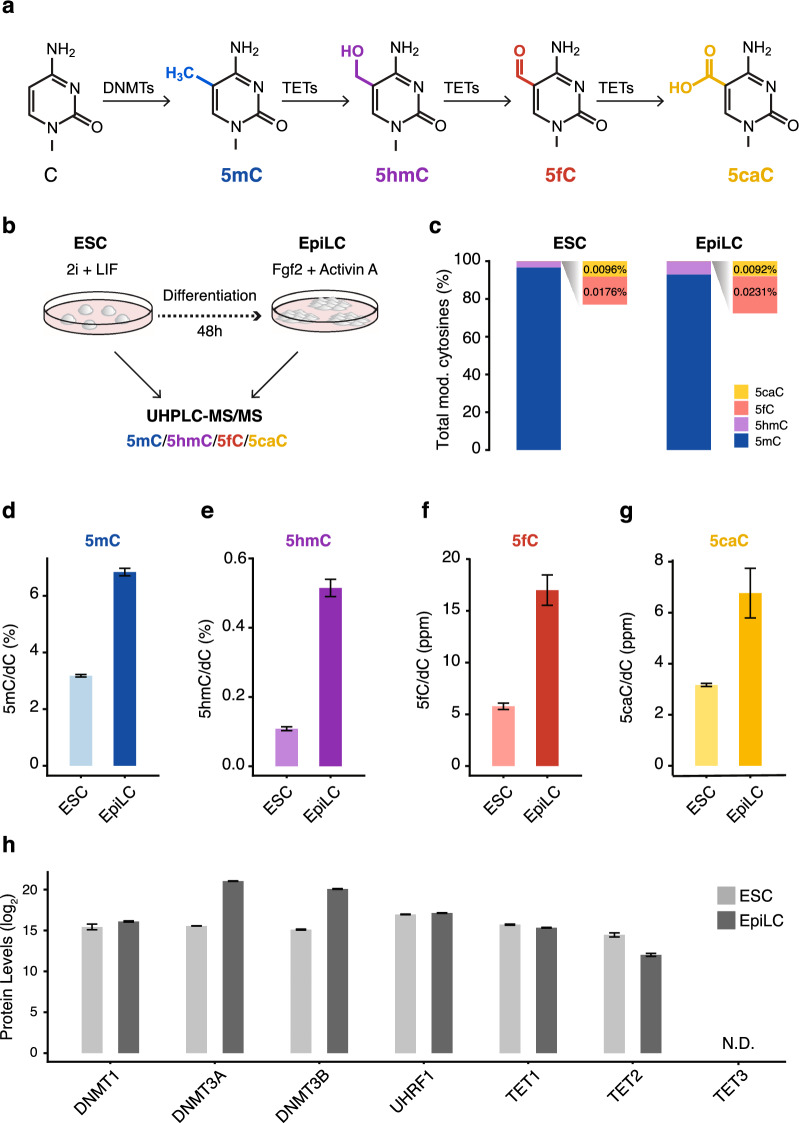
Global increases in cytosine modifications accompany the transition from naive to primed pluripotency. (**a**) Cytosine modifications depicted with the enzymes responsible for their generation. (**b**) Schematic overview of experimental design. DNA modifications were measured in murine naive embryonic stem cells (ESC) and epiblast-like stem cells (EpiLC) using UHPLC-MS/MS. (**c**) Abundance of genomic 5mC, 5hmC, 5fC, and 5caC in wild-type ESCs and EpiLCs shown as the fraction of total modified (mod.) cytosines. Due to their relative scarcity, 5fC and 5caC are depicted with a zoomed-in view. *n* = 6 (ESCs) and *n* = 12 (EpiLCs) biological replicates. (**d**–**g**) Global levels of (**d**) 5mC, (**e**) 5hmC, (**f**) 5fC, and (**g**) 5caC in wild-type ESCs and EpiLCs as determined by mass spectrometry (UHPLC-MS/MS). DNA modification levels are expressed as a percentage (%) or parts per million (ppm: 1 ppm = 0.0001%) of total cytosine (dC). Error bars indicate mean ± SD calculated from *n* = 6 (ESCs) and *n* = 12 (EpiLCs) biological replicates. (**h**) Protein abundance of DNA modifying enzymes in wild-type ESCs and EpiLCs as determined by LC–MS/MS-based whole proteome profiling. Shown are log2-transformed protein levels. Error bars indicate mean ± SD calculated from *n* = 3 (ESCs) and *n* = 3 (EpiLCs) biological replicates. N.D.: no peptides of protein detected.

The cellular landscape of 5mC can be altered by the inhibition of maintenance DNA methylation and/or via the action of the Ten-eleven Translocation (TET) family of dioxygenases^11^. The three mammalian homologs, TET1, TET2, and TET3, share a conserved dioxygenase domain and catalyze the stepwise oxidation from 5mC to 5-hydroxymethylcytosine (5hmC), 5-formylcytosine (5fC), and 5-carboxylcytosine (5caC) (Fig. 1a)^12–15^. These oxidized cytosine derivatives have been described as intermediates of passive and active DNA demethylation^14,16–18^, yet may also serve as stable epigenetic marks^19,20^. Moreover, their largely separate genomic distributions and reader proteins imply distinct epigenetic regulatory functions for 5hmC, 5fC, and 5caC^21,22^.

TET-mediated cytosine oxidation is indispensable for mammalian development^23–26^, as evidenced by the failure of TET-deficient mice to develop beyond gastrulation^25,26^. However, single *Tet* mutants exhibit less severe albeit distinct phenotypes, suggesting each enzyme can partially compensate for loss of the other^27–29^. While all TETs oxidize 5mC, the three TETs are not entirely functionally redundant. Individual TET family members exhibit distinct cellular localization patterns and genome-wide binding profiles, which appear to confer them with discrete functions during development^30–32^.

Despite extensive research into the differing functions of TETs, the precise roles of the three TET proteins in the stepwise oxidation of 5mC in vivo remains to be elucidated. Clearly, the observed stable cellular levels of oxidized cytosine derivatives and their distinct genome-wide distributions seem to require dedicated regulatory mechanisms for each oxidation step^19–21,33^. Interestingly, the three TET proteins differ in their large, unstructured N-terminal domains, possibly enabling divergent contributions to stage and cell-type specific DNA modification^12^. While TET1/2/3 have all been demonstrated to mediate iterative cytosine oxidation in vitro, whether these proteins equally contribute to the levels of the three oxidized cytosine derivatives in a cellular context is unclear^13,14^. Moreover, currently available biochemical data do not conclusively resolve whether TET proteins oxidize 5mC in a chemically processive manner or in a rather distributive mode with independent steps^34–36^.

Due to fast kinetics and limited material, studying the dynamics of DNA modifications during mammalian peri-implantation development remains experimentally intractable. The naive pluripotent state of the pre-implantation mouse embryo can be captured and maintained in vitro by culturing murine embryonic stem cells (ESCs) in the presence of leukemia inhibitory factor (LIF) and inhibitors of MEK and GSK3 (2i)^37^. These naive ESCs feature closely similar transcriptional and epigenetic characteristics of the E3.75-E4.5 ICM from which they are derived^38^, including global DNA hypomethylation^39–41^. The transition from naive to primed pluripotency accompanying peri-implantation development can be recapitulated in vitro by differentiating naive ESCs into epiblast-like cells (EpiLCs) by exposure to fibroblast growth factor 2 and Activin A. After 48 h of differentiation, EpiLCs exhibit both a transcriptional profile and genome-wide DNA hypermethylation that closely resembles that of the post-implantation pre-gastrulation epiblast (E5.75-E6.5)^10,42,43^. As such, this in vitro system offers an ideal model for uncovering basic principles of oxidized cytosine regulation.

Here, we combine quantitative proteomics and global DNA modification measurements to dissect the individual contributions of TET enzymes to cytosine modification dynamics during the transition from naive to primed pluripotency. We find that TET1 and TET2 distinctly contribute to global oxidized cytosine levels in naive ESCs as well as EpiLCs. While TET2 is required for the formation of 5hmC in the naive state, TET1 is responsible for most of the global 5hmC wave during the transition to primed pluripotency. Most notably, despite a strong downregulation during differentiation, TET2 accounts for the majority of 5fC in both stages of pluripotency.

## Results

We first set out to characterize DNA modification dynamics in the naive to primed transition. To this end, we used ultra-high performance liquid chromatography coupled to tandem mass spectrometry (UHPLC-MS/MS) to quantitatively assess the levels of 5mC, 5hmC, 5fC, and 5caC in genomic DNA isolated from wild-type (wt) mouse naive ESCs and EpiLCs differentiated for 48 h (Fig. 1b). As previous studies have shown^12–15^, we found that cytosine modifications become exceedingly less abundant with higher oxidation states. In ESCs 5hmC constitutes ~ 3% of modified cytosines whereas 5fC and 5caC make up only 0.02% and 0.01%, respectively. In EpiLCs, a similar distribution is observable, albeit with 5hmC and 5fC accounting for a larger fraction of modified cytosines than in ESCs (Fig. 1c).

Global DNA methylation (5mC) increased over the course of differentiation with 5mC levels in naive ESCs and EpiLCs reminiscent of those in their respective in vivo counterparts, the E3.5 ICM and E6.5 epiblast^6, 8^ (Fig. 1d; Supplementary Table S1). The precise quantification of cytosine derivatives demonstrated that, along with 5mC, the levels of 5hmC, 5fC, and 5caC increased from ESCs to EpiLCs (Fig. 1e–g; Supplementary Table S1). While 5mC and 5caC levels doubled, 5hmC and 5fC displayed a five- and three-fold increase, respectively. This overproportional increase of 5hmC and 5fC suggests that the oxidation of 5mC may occur in successive steps subjected to independent regulation during exit from naive pluripotency.

In search of possible mechanisms for the uncoupled levels of cytosine derivatives, we examined the protein abundance of cytosine modifying enzymes (DNMTs and TETs) during the naive to primed transition. Mass spectrometry (MS)-based quantitative proteomics showed the global wave of DNA methylation during differentiation to coincide with a substantial increase in the levels of the de novo DNA methyltransferases DNMT3A and DNMT3B (Fig. 1h; Supplementary Table S2), consistent with similar changes observed during peri-implantation development^9,44,45^. Protein levels of the ubiquitous maintenance DNA methyltransferase DNMT1 as well as its essential regulator and cofactor UHRF1 remained constant in the naive to primed transition (Fig. 1h). Despite even larger gains in oxidized cytosine levels, we did not detect corresponding increases in TET protein levels during the transition from naive ESCs to EpiLCs. On the contrary, while TET1 levels remained relatively constant, we measured a significant reduction (~ 4.5 fold) in TET2 peptides in EpiLCs and failed to detect TET3 in either cell type (Fig. 1h). These changes in TET protein levels were directly confirmed by independent Western blot analyses and reflected similar trends in *Tet* mRNA levels as determined by qPCR (Supplementary Fig. S1a,b). Moreover, these data are consistent with the expression profile of TETs during in vivo peri-implantation development, where TET1 and TET2 but not TET3 are expressed^46–48^.

As the overall abundance of TET family members decreases during the naive to primed transition, we considered whether the increase in oxidized cytosine derivatives might be attributable to expression changes in the Base Excision Repair (BER) pathway. Genomic 5fC and 5caC can be specifically recognized and excised by thymine DNA glycosylase (TDG), and ultimately replaced by unmodified cytosine via the BER pathway^49^. As such, the abundance of modified cytosines, especially 5fC and 5caC, in genomic DNA is subject to influence from the BER pathway^50^. However, our proteomics data from ESCs and EpiLCs indicated that levels of the BER proteins (e.g. APEX1, LIG3, PNKP, XRCC1, and PARP1) remained largely unchanged (Supplementary Fig. S1c). To assess the expression of additional BER factors undetected in our proteomics analysis, we profiled the transcriptomes of ESCs and EpiLCs using RNA-seq (Supplementary Table S3). In line with our proteomics measurements, most BER genes exhibited mostly static transcript levels in the naive to primed transition, whereas the expression of *Tdg* even increased (Supplementary Fig. S1c). These data argue against reduced removal of oxidized cytosine derivatives by the BER pathway as an explanation for the observed increase in 5hmC and 5fC levels during the naive to primed pluripotency transition. Additionally, we assessed the expression profile of factors involved in alternative base modification pathways, such as the AID/APOBEC family of cytosine deaminases. However, we failed to detect the majority of these deaminases, including AID (AICDA), in either our proteome or transcriptome data from ESCs and EpiLCs (Supplementary Fig. S1c). Together with our previous work demonstrating the deamination pathway to negligibly influence 5hmC levels in ESCs^51^, these results indicate that the AID/APOBEC enzymes do not appreciably contribute to the global increase in oxidized cytosine levels in the transition from naive to primed pluripotency.

**Figure 2:**
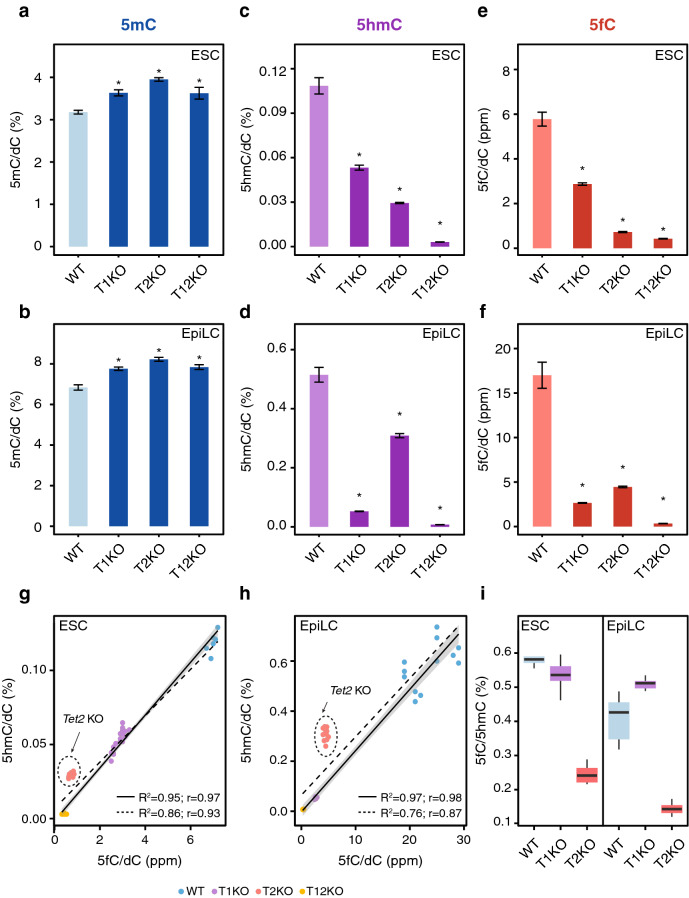
Quantification of cytosine modifications in *Tet1* and *Tet2* knockout ESCs and EpiLCs. (**a**–**f**) Global levels of (**a**, **b**) 5mC, (**c**, **d**) 5hmC, and (**e**, **f**) 5fC, in wild-type (WT), *Tet1* KO (T1KO), *Tet2* KO (T2KO), and *Tet1/Tet2* DKO (T12KO) ESCs and EpiLCs as determined by mass spectrometry (UHPLC-MS/MS). DNA modification levels are expressed as a percentage (%) or parts per million (ppm: 1 ppm = 0.0001%) of total cytosine (dC) and shown as the mean ± SD of biological replicates as follows: WT (ESCs: *n* = 18; EpiLCs: *n* = 24), T1KO (ESCs: *n* = 18; EpiLCs: *n* = 12), T2KO (ESCs: *n* = 12; EpiLCs: *n* = 12), and T12KO (ESCs: *n* = 12; EpiLCs: *n* = 12). * p < 0.005 to wt as determined using a one-way ANOVA followed by a post-hoc Tukey HSD test. (**g**–**h**) Correlations between 5hmC and 5fC levels in wt and *Tet* KO (**g**) ESCs and (**h**) EpiLCs. The dashed regression line was generated using the full data set, the solid regression line was generated by excluding *Tet2* KO data. Depicted are values from the individual replicates presented in **c**–**f**. R^2^: coefficient of determination; r: Pearson correlation coefficient. (**i**) Box plots of the ratio of 5fC to 5hmC in wt, *Tet1* KO and *Tet2* KO ESCs and EpiLCs. Unlike the *Tet1* KO, *Tet2* KO drastically affects the 5fC/5hmC ratio. The median is represented by the central bold line. The lower and upper hinges correspond to the first and third quartiles (the 25th and 75th percentiles). The upper and lower whisker extend from the hinge to the largest and lowest value, respectively, no further than 1.5 * interquartile range (IQR).

We next sought to dissect and identify the specific contributions of TET proteins to cytosine modifications during the naive to primed transition. To this end, we used CRISPR/Cas-mediated mutagenesis to generate *Tet1* and *Tet2* single knockout (KO) and *Tet1/Tet2* double KO (DKO) ESC lines (Supplementary Fig. S2a,b) and confirmed loss of TET1 and TET2 by Western blot analyses (Supplementary Fig. S2c,d). Using two independent clones for each genotype, we quantified the levels of 5mC, 5hmC, 5fC, and 5caC in ESCs and EpiLCs by LC–MS/MS. In parallel, we used RNA-seq and MS-based proteomics to monitor how loss of TET1 and/or TET2 affected the transcriptome and proteome of ESCs and EpiLCs (Supplementary Table S2). Elimination of either TET1 or TET2, or both TET1 and TET2 resulted in modest yet significant increases in 5mC in both naive ESCs and primed EpiLCs (Fig. 2a,b; Supplementary Table S1 and S4). The expression levels of DNMT1, DNMT3A/B, and UHRF1 in *Tet* KO ESCs and EpiLCs were similar to those in their wild-type counterparts, suggesting the 5mC gains were not a result of upregulated DNA methylating enzymes (Supplementary Fig. S3). Double *Tet1/Tet2* KO resulted in the loss of practically all oxidized cytosine derivatives, with levels of 5hmC, 5fC, and 5caC reduced to near or below the detection limit in ESCs and EpiLCs (Fig. 2c–f; Supplementary Fig. S4a, b; Supplementary Table S1 and S4). Together with our expression data (Fig. 1h; Supplementary Fig. S1a,b and S3) these results argue for major roles of TET1 and TET2 in 5mC oxidation during naive pluripotency exit with little to no contribution from TET3.

Analysis of the individual *Tet* KOs revealed stark, stage-specific differences in each enzyme’s functional contribution to the consecutive steps of cytosine oxidation. Genomic 5hmC levels were significantly decreased in *Tet1* KO (50% of wt 5hmC) as well as *Tet2* KO (30% of wt 5hmC) ESCs demonstrating that, despite both being highly expressed, TET1 and TET2 are not redundant (Fig. 2c; Supplementary Table S4). The comparatively severe 5hmC depletion in *Tet2* KO ESCs indicates the majority of 5mC to 5hmC conversion in naive pluripotency to require TET2. Strikingly, *Tet2* KO 5hmC levels substantially increased upon exit from pluripotency, recovering from ~ 0.03% (30% of wt ESC 5hmC) to ~ 0.3% of genomic cytosines (60% of wt EpiLC 5hmC). As 5hmC increases in the absence of TET2 in *Tet2* KO EpiLCs, this suggests that the majority of 5hmC newly acquired during differentiation is generated by TET1, which remains highly expressed in EpiLCs (Fig. 2c,d, Supplementary Fig. S3, Supplementary Table S1 and S4). Supporting this notion was the finding that *Tet1* KOs fail to acquire 5hmC upon exit from naive pluripotency, with 5hmC levels remaining essentially unchanged between naive and primed pluripotency (~ 0.05% in *Tet1* KO ESCs and ~ 0.06% in *Tet1* KO EpiLCs versus ~ 0.5% of genomic cytosines in wt EpiLCs) (Fig. 2c,d and Supplementary Table S1).

Notably, EpiLCs express TET1 at levels similar to naive ESCs (Fig. 1h) and possess higher 5hmC levels (~ 0.5% versus ~ 0.1% of genomic cytosines). However, TET1, even in the absence of TET2 (in *Tet2* KO), is able to generate 60% of cellular 5hmC in EpiLCs (Fig. 2c, d; Supplementary Table S1 and S4). In other words, comparable amounts of TET1 produce ten-times more 5hmC in EpiLCs versus ESCs (~ 0.3% versus ~ 0.03% of genomic cytosines in *Tet2* KOs). Taken together, TET1 and TET2 possess distinct, stage-specific roles in the oxidation of 5mC, in which the responsibility of 5hmC formation passes from TET2 to TET1 upon differentiation.

To investigate whether similar stage-dependent preferences apply for the subsequent oxidation step, i.e. the conversion of 5hmC to 5fC, we compared 5fC levels in ESCs and EpiLCs. Analysis of 5fC levels in KO lines revealed an unexpectedly prominent role of TET2 in ESCs and even EpiLCs (Fig. 2e,f; Supplementary Table S1 and S4). In naive ESCs, *Tet2* KO caused an ~ 87% reduction in 5fC levels, almost reaching the background levels of the *Tet1/Tet2* DKO, whereas only 50% of 5fC was lost in *Tet1* KO ESCs (Fig. 2e). As the reduction of 5fC in *Tet1* KO ESCs was proportional to the loss of its precursor, 5hmC, the overall 5fC/5hmC ratio remained similar to that of wild-type ESCs (Fig. 2g, i). In striking contrast, the large reduction of 5fC in naive *Tet2* KO ESCs did not correlate with a decrease in 5hmC, with TET2 loss leading to a much lower ratio of 5fC/5hmC than in wt or *Tet1* KO ESCs (Fig. 2g, i). Thus, TET2 is required for the majority of global cytosine oxidation in naive pluripotency, with TET1 unable to compensate for TET2 loss in naive ESCs.

In EpiLCs, 5fC levels dropped to ~ 18% and ~ 26% of their wt levels in *Tet1* KO and *Tet2* KO cells, respectively (Fig. 2f). The similarity of 5fC levels in both, *Tet1* and *Tet2* KO EpiLCs stands in stark contrast to their 5hmC levels (Fig. 2d). As in naive ESCs, the stark reduction of 5fC in *Tet1* KO EpiLCs was accompanied by a strong decrease in 5hmC. However, the loss of TET2 in EpiLCs led to a disproportionate decrease in 5fC compared to 5hmC (Fig. 2g–i). The significant global depletion of 5fC resulting from TET2 loss in EpiLCs is particularly striking considering that TET2 is expressed at lower levels at this particular stage compared to ESCs (Fig. 1h, Supplementary Fig. S1a).

As 5fC can be excised from DNA by the BER pathway, we investigated whether the decrease in 5fC in *Tet2* KOs might be an indirect consequence resulting from upregulation of DNA repair enzymes upon TET2 loss. We assessed the expression levels of BER pathway proteins by RNA-seq and full proteome mass spectrometry at both time points (Supplementary Fig. S3). Neither the loss of TET2 nor TET1 significantly affected the expression of these genes in ESCs or EpiLCs. Therefore, the decrease of 5fC in *Tet2* KOs appears to be a direct effect of TET2 loss. The disproportionate loss of 5fC in both stages, naive and primed, reveals a previously unappreciated prominence of TET2 in the formation of 5fC in pluripotent stem cells.

Due to the extremely low abundance of 5caC in comparison to the other cytosine modifications (Fig. 1c,g), loss of TET activity resulted in levels below the detection limit (Supplementary Fig. S4). We were only able to clearly detect 5caC in wt and *Tet2* KO EpiLCs, but not *Tet1* KO EpiLCs, suggesting that the more abundant TET1 is responsible for most 5caC formation in EpiLCs.

## Discussion

**Figure 3:**
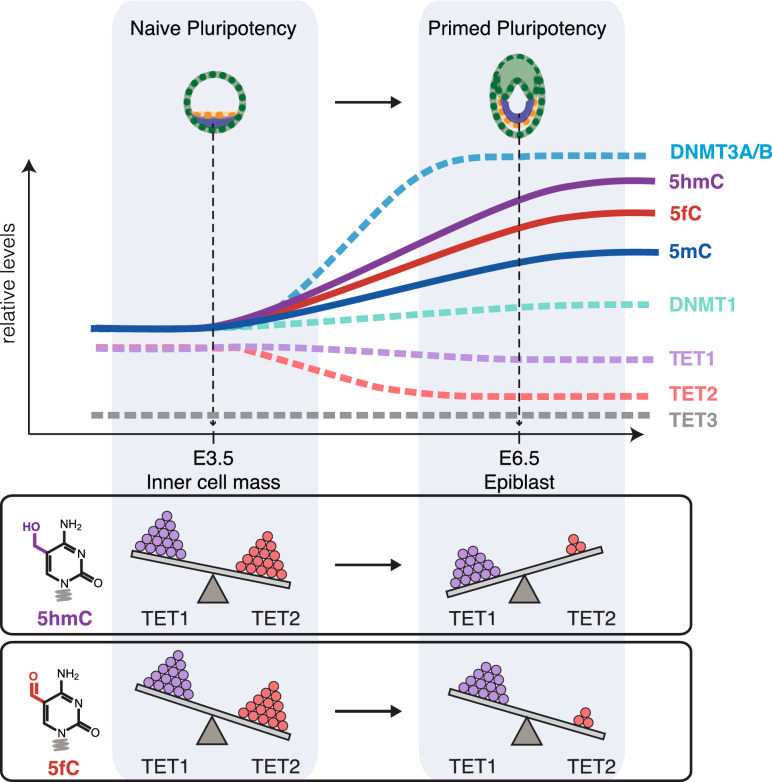
Epigenetic changes and distinct contributions of different DNA modifying enzymes during the transition from naive to primed pluripotency. Graphical summary depicting changes in cellular levels of cytosine modifications and their respective DNA modifying enzymes in the transition from naive to primed pluripotency. The relative contributions of TET1 and TET2 to the generation of 5hmC and 5fC as estimated from observations in *Tet* KO ESCs and EpiLCs are illustrated at the bottom; the number of spheres and tilt of the balance represent the protein abundance of each TET and the contribution of each TET to the levels of the depicted cytosine derivative, respectively. TET1 gains importance in the oxidation of 5mC to 5hmC during differentiation as TET2 abundance decreases. Most remarkably, despite drastic downregulation TET2 remains critical for the formation of 5fC in primed pluripotency.

In summary, the systematic quantification of cytosine derivatives and their respective enzymes in this defined cellular differentiation system leads to a number of unexpected findings (Fig. 3). Whereas the increase of 5mC during naive pluripotency exit correlated with the growing abundance of the de novo DNA methyltransferases, DNMT3A and DNMT3B, the rising levels of oxidized cytosine derivatives, 5hmC and 5fC, were accompanied by stable TET1 and diminishing TET2 levels. In these cells, TET3 seems to play little to no role given its undetectable expression and the practically complete loss of genomic 5hmC, 5fC, and 5caC in cells lacking TET1 and TET2.

Our analysis of global cytosine modification levels in *Tet1* and *Tet2* KO ESCs and EpiLCs revealed both enzymes to have profound stage-specific contributions to cytosine oxidation, which cannot be fully compensated by the other enzyme. In ESCs, the oxidation of 5mC to 5hmC relies primarily on TET2, whereas the global increase in 5hmC during differentiation is almost exclusively catalyzed by TET1. Thus, the distinct, stage-specific contributions of TET1 and TET2 to 5hmC generation might underlie their opposing roles in controlling the transition between naive and primed pluripotency^52^.

The previously observed downregulation had argued against any role of TET2 in peri-implantation development^28,53^. We also observed downregulation of *Tet2* expression in EpiLCs but still detected TET2 protein by mass spectrometry and Western blot analysis. In fact, our KO data identified a rather distinct role of TET2 in naive and primed pluripotency. Remarkably, *Tet2* KO ESCs and EpiLCs show an unexpected loss of 5fC, arguing that TET2 governs the formation of 5fC in ESCs as well as the increase of 5fC during the naive to primed transition. Our KO data clearly demonstrate that the residual amounts of TET2 proteins in EpiLCs have a prominent role in the oxidation of 5hmC to 5fC, which cannot be compensated by the much more abundant TET1.

Detailed analysis of the different KO lines also showed that 5hmC and 5fC levels respond independently. Since TET1 can rescue the majority of 5hmC but not 5fC upon loss of TET2 (especially in EpiLCs), we propose that in vivo stepwise oxidation largely follows a distributive model in line with previous in vitro findings^35,36^ with the caveat that the in vivo distributive oxidation is shaped by an additional layer of regulation, one in which different TET paralogs preferentially catalyze separate steps. Our results suggest TET1 preferentially oxidizes 5mC to 5hmC, then dissociates, leaving the subsequent oxidation step of 5hmC to 5fC to be catalyzed by TET2. In line with this hypothesis, a similar division between TET1 and TET2 activities has been described for SALL4A-bound enhancers^32^.

In addition to the apparently differing substrate proclivities of TET1 and TET2, we observe a differentiation-dependent, dynamic regulation of oxidative potential, especially for TET1. Despite maintaining comparable protein levels in the naive to primed transition, TET1 drives the differentiation-dependent quintupling of 5hmC almost exclusively and independent of TET2, yet can only contribute to 30% of 5hmC in naive ESCs.

Thus, not only the underlying mechanisms regulating the individual TET-specific contribution to distributive oxidation deserve further investigation, but also those controlling the dynamics of substrate oxidation. It remains to be seen to what extent modulation of the catalytic activity of the three TET enzymes by differential isoform expression, posttranslational modifications, interacting factors, and site-specific recruitment could constitute an additional layer of epigenetic regulation. Interestingly, 5fC was revealed to possess novel characteristics, such as the ability to distort the DNA double helix^54^ and directly mediate DNA–protein crosslinks^55,56^, with potentially far reaching consequences on transcriptional regulation and chromatin remodeling^57,58^. In this context, our observation that 5fC formation appears to be largely TET2-dependent might also have novel implications for understanding how *Tet2* mutations contribute to cancerogenesis.

## Methods

### Cell culture

Naive J1 mESCs were cultured and differentiated into EpiLCs as described previously^59,60^. In brief, for both naive ESCs and EpiLCs defined media was used, consisting of: N2B27 (50% neurobasal medium (Life Technologies), 50% DMEM/F12 (Life Technologies)), 2 mM l-glutamine (Life Technologies), 0.1 mM β-mercaptoethanol (Life Technologies), N2 supplement (Life Technologies), B27 serum-free supplement (Life Technologies), and 100 U/mL penicillin, 100 μg/mL streptomycin (Sigma). Naive ESCs were maintained on flasks treated with 0.2% gelatin in defined media containing 2i (1 μM PD032591 and 3 μM CHIR99021 (Axon Medchem, Netherlands)), 1,000 U/mL recombinant leukemia inhibitory factor (LIF, Millipore), and 0.3% BSA (Gibco) for at least three passages before commencing differentiation.

For CRISPR-assisted cell line generation mESCs were maintained on 0.2% gelatin-coated dishes in Dulbecco's modified Eagle's medium (Sigma) supplemented with 16% fetal bovine serum (FBS, Biochrom), 0.1 mM ß-mercaptoethanol (Invitrogen), 2 mM l-glutamine (Sigma), 1 × MEM Non-essential amino acids (Sigma), 100 U/mL penicillin, 100 μg/mL streptomycin (Sigma), homemade recombinant LIF tested for efficient self-renewal maintenance, and 2i (1 μM PD032591 and 3 μM CHIR99021 (Axon Medchem, Netherlands)).

To differentiate naive ESCs into epiblast-like cells, cells were plated on flasks treated with Geltrex (Life Technologies) diluted 1:100 in DMEM/F12 (Life Technologies) in defined medium containing 10 ng/mL Fgf2 (R&D Systems), 20 ng/mL Activin A (R&D Systems) and 0.1 × Knockout Serum Replacement (KSR) (Life Technologies). Media was changed after 24 h and EpiLCs were harvested after 48 h.

Cells were regularly tested for Mycoplasma contamination by PCR.

### CRISPR/Cas-mediated gene knockout and Western blot

For the generation of *Tet1* and *Tet2* knockouts, *Tet1* and *Tet2*-specific gRNAs (Supplementary Table S3) were cloned into puromycin-selectable vector expressing both SpCas9 and gRNA (px459: F. Zhang Lab). mESCs were transfected with Cas9-gRNA vectors using Lipofectamine 3000 (Invitrogen) according to manufacturer’s protocol. Two days after transfection, J1 mESCs were plated at clonal density in ESC media supplemented with 1 µg/mL puromycin (Gibco). Selection media was removed after 48 h, replaced with normal ESC media, and colonies were allowed to grow for an additional 4–5 days. Single ESC colonies were transferred into 96-well plates and the plates were duplicated after 2 days. Enrichment for mutated clones was accomplished by amplifying the CRISPR/Cas targeted region via PCR (oligonucleotides in Supplementary Table S5) and performing restriction-fragment length polymorphism (RFLP) analysis^61^ with SacI or EcoRV (FastDigest; Thermo Scientific) for *Tet1* or *Tet2*, respectively (see also Supplementary Fig. S2a). Cell lysis in 96-well plates, PCR on lysates, and restriction digest were performed as previously described^60^.

Clones harboring biallelic mutations were then assessed for loss of TET1 or TET2 via Western blot. Western blots for both *Tet* KOs were performed as described previously^60^ using monoclonal antibodies (rat anti-TET1 5D6, rat anti-TET2 9F7, and rat anti-TET3 23B9)^62^ and polyclonal rabbit anti-H3 (ab1791, Abcam) as loading control. Blots were probed with secondary antibodies anti-rat (112-035-068, Jackson ImmunoResearch) and anti-rabbit (170–6515, Bio-Rad) conjugated to horseradish peroxidase (HRP) and visualized using an ECL detection kit (Thermo Scientific Pierce).

### Quantitative real-time PCR (qRT-PCR) Analysis

Total RNA was isolated using the NucleoSpin Triprep Kit (Macherey-Nagel) according to the manufacturer's instructions. cDNA synthesis was performed with the High-Capacity cDNA Reverse Transcription Kit (with RNase Inhibitor; Applied Biosystems) using 500 ng of total RNA as input. Oligonucleotides used in qRT-PCR assays are listed in Supplementary Table S5 were performed in 10 µL reactions with 5 ng of cDNA used as input. For TaqMan and SYBR green detection, TaqMan Universal Mastermix (Applied Biosystems) and FastStart Universal SYBR Green Master Mix (Roche) were used, respectively. The reactions were run on a LightCycler480 (Roche).

### RNA-seq

Digital gene expression libraries for RNA-seq were prepared using the single-cell RNA barcoding sequencing (SCRB-seq) method as described previously^63–65^, with minor modifications to accommodate bulk cell populations. In brief, RNA was extracted and purified from ~ 1 × 10^6^ cells using the NucleoSpin Triprep Kit (Machery-Nagel) according to the manufacturer’s instructions. In the initial cDNA synthesis step, purified, bulk RNA (70 ng) from individual samples were subjected to reverse transcription in 10 μL reactions containing 25 units of Maxima H Minus reverse transcriptase (ThemoFisher Scientific), 1 × Maxima RT Buffer (ThemoFisher Scientific), 1 mM dNTPs (ThermoFisher Scientific), 1 µM oligo-dT primer with a sample-specific barcode (IDT), and 1 µM template-switching oligo (IDT). Reverse transcription reactions were incubated 90 min at 42 °C. Next, the barcoded cDNAs from individual samples were pooled together and then purified using the DNA Clean & Concentrator-5 Kit (Zymo Research) according to the manufacturer's instructions. Purified pooled cDNA was eluted in 18 μL DNase/RNase-Free Distilled Water (Thermo Fisher) and then, to remove residual primers, incubated with 1 μL Exonuclease I Buffer (NEB) and 1 μL Exonuclease I (NEB) (final reaction volume: 20 μL) at 37 C for 30 min followed by heat-inactivation at 80 C for 20 min. Full-length cDNA was then amplified via PCR using KAPA HiFi HotStart ReadyMix (KAPA Biosystems) and SINGV6 primer (IDT). The pre-amplification PCR was performed using the following conditions: 3 min at 98 °C for initial denaturation, 10 cycles of 15 s at 98 °C, 30 s at 65 °C, and 6 min at 68 °C, followed by 10 min at 72 °C for final elongation. After purification using CleanPCR SPRI beads (CleanNA), the pre-amplified cDNA pool concentration was quantified using the Quant-iT PicoGreen dsDNA Assay Kit (Thermo Fisher). A Bioanalzyer run using the High-sensitivity DNA Kit (Agilent Technologies) was then performed to confirm the concentration and assess the size distribution of the amplified cDNA pool (Agilent Technologies). Next, 0.8 ng of the pure, amplified cDNA pool was used as input for generating a Nextera XT DNA library (Illumina) following the Manufacturer’s instructions with the exception that a custom P5 primer (P5NEXTPT5) (IDT) was used to preferentially enrich for 3′ cDNA ends in the final Nextera XT Indexing PCR^63–65^. After an initial purification step using a 1:1 ratio of CleanPCR SPRI beads (CleanNA), the amplified Nextera XT Library the 300–800 bp range of the library was size-selected using a 2% E-Gel Agarose EX Gels (Life Technologies) and then extracted from the gel using the MinElute Gel Extraction Kit (Qiagen, Cat. No. 28606) according to manufacturer’s recommendations. The final concentration, size distribution, and quality of Nextera XT library were assessed with a Bioanalyzer (Agilent Technologies) using a High-sensitivity DNA Kit (Agilent Technologies). The Nextera XT RNA-seq library was paired-end sequenced using a high output flow cell on an Illumina HiSeq 1500. In read 1, sample-specific barcodes were obtained by sequencing the first 16 bases, while the sequence of the cDNA fragment was obtained by the 50 bases in read 2. An additional 8 base i7 barcode read was performed to distinguish the library from others sequenced in parallel on the same flow cell.

### RNA-seq processing and analysis

Raw RNA-seq data was processed and mapped to the mouse genome (mm10) using the zUMIs pipeline^66^. Gene annotations were obtained from Ensembl (GRCh38.84 or GRCm38.75). UMI count tables were filtered for low counts using HTSFilter^67^. Differential expression analysis was performed in R using DESeq2^68^ and genes with an adjusted *P* < 0.05 were considered to be differentially expressed.

### UHPLC-MS/MS analysis of DNA samples

#### DNA digestion

Isolation of genomic DNA was performed according to earlier published work^51^.

1.0–5 μg of genomic DNA in 35 μL H_2_O were digested as follows: an aqueous solution (7.5 μL) of 480 μM ZnSO_4_, containing 18.4 U nuclease S1 (Aspergillus oryzae, Sigma-Aldrich), 5 U Antarctic phosphatase (New England BioLabs) and labeled internal standards were added ([^15^N_2_]-cadC 0.04301 pmol, [^15^N_2_,D_2_]-hmdC 7.7 pmol, [D_3_]-mdC 51.0 pmol, [^15^N_5_]-8-oxo-dG 0.109 pmol, [^15^N_2_]-fdC 0.04557 pmol) and the mixture was incubated at 37 °C for 3 h. After addition of 7.5 μl of a 520 μM [Na]_2_-EDTA solution, containing 0.2 U snake venom phosphodiesterase I (Crotalus adamanteus, USB corporation), the sample was incubated for 3 h at 37 °C and then stored at − 20 °C. Prior to LC/MS/MS analysis, samples were filtered by using an AcroPrep Advance 96 filter plate 0.2 μm Supor (Pall Life Sciences).

#### UHPLC-MS/MS analysis

Quantitative UHPLC-MS/MS analysis of digested DNA samples was performed using an Agilent 1290 UHPLC system equipped with a UV detector and an Agilent 6490 triple quadrupole mass spectrometer. Natural nucleosides were quantified with the stable isotope dilution technique. An improved method, based on earlier published work^51,69^ was developed, which allowed the concurrent analysis of all nucleosides in one single analytical run. The source-dependent parameters were as follows: gas temperature 80 °C, gas flow 15 L/min (N_2_), nebulizer 30 psi, sheath gas heater 275 °C, sheath gas flow 15 L/min (N_2_), capillary voltage 2,500 V in the positive ion mode, capillary voltage − 2,250 V in the negative ion mode and nozzle voltage 500 V. The fragmentor voltage was 380 V/ 250 V. Delta EMV was set to 500 V for the positive mode. Compound-dependent parameters are summarized in Supplementary Table S6. Chromatography was performed by a Poroshell 120 SB-C8 column (Agilent, 2.7 μm, 2.1 mm × 150 mm) at 35 °C using a gradient of water and MeCN, each containing 0.0085% (v/v) formic acid, at a flow rate of 0.35 mL/min: 0 → 4 min; 0 → 3.5% (v/v) MeCN; 4 → 6.9 min; 3.5 → 5% MeCN; 6.9 → 7.2 min; 5 → 80% MeCN; 7.2 → 10.5 min; 80% MeCN; 10.5 → 11.3 min; 80 → 0% MeCN; 11.3 → 14 min; 0% MeCN. The effluent up to 1.5 min and after 9 min was diverted to waste by a Valco valve. The autosampler was cooled to 4 °C. The injection volume amounted to 39 μL. Data were processed according to earlier published work^51^.

### MS-based quantitative proteomics

#### Full proteome sample preparation

For full proteome measurements flash-frozen cells were lysed in 200 µL of the lysis buffer (6 M Guanidinium Chloride, 100 mM Tris–HCl pH 8.5 and freshly added 2 mM DTT). By thoroughly pipetting, samples were homogenized and subsequently boiled at 99 °C for 10 min in a thermal shaker at 1,700 rpm. To get rid of bubbles and to collect the evaporated liquid, samples were quickly spun down. After sonication for 15 min (30 s on/off interval, Bioruptor Plus by Diagenode) protein concentrations were estimated by a BCA assay in a TECAN reader. Chloroacetamide was added to the samples (40 mM final concentration) and samples were incubated at room temperature for 20 min. For the protein digestion, 30 µg of the lysate was diluted in 30 µL of the lysis buffer already including 2 mM DTT and 40 mM CAA. Then, samples were diluted 1:10 in the digestion buffer (25 mM Tris–HCl pH 8.5 and 10% acetonitrile) containing trypsin and LysC in a 1:50 protease to protein ratio. Digestion was performed overnight at 37 °C and 100 rpm. After acidifying samples with 1% trifluoroacetic acid (TFA), peptides were cleaned up on three layers of SDB-RPS matrix^70^. Eluted and speedvac dried peptides were resuspended in 20 µL of A* buffer (0.1% TFA and 2% acetonitrile) and peptide concentrations were estimated by nanodrop at 280 nm.

#### Full proteome measurements based on data-independent acquisition method

Mass spectrometric analysis of peptides was performed on a quadrupole Orbitrap mass spectrometer (Q Exactive HF-X, ThermoFisher Scientific, Bremen, Germany) after prior nanoflow liquid chromatography on an Easy-nLC 1200 (ThermoFisher Scientific). The injection was mediated under high-pressure conditions via a nano-electrospray ion source. For this purpose, in-house packed 50 cm columns of ReproSil-Pur C18-AQ 1.9-µm resin (Dr. Maisch GmbH) were used to elute approximately 400 ng peptides of each sample in an acetonitrile gradient for 120 min. The flow rate was kept constantly at around 300 nL/min and the column oven temperature at 60 °C.

The peptides were analyzed following a data-independent acquisition (DIA) method (MS1 scan: resolution 60,000, 300 to 1,650 m/z, maximum injection time 60 ms and AGC target 3E6, MS2 scan: resolution 30,000, 32 segments at varying isolation windows ranging from 14.4 m/z to 562.8 m/z, maximum injection time 54 ms and AGC target 3E6). For MS2 scans the default charge state was set to 2. The stepped normalized collision energy was set to 25, 27.5 and 30.

#### Processing of DIA data

The DIA raw files were analyzed with the Spectronaut Pulsar X software package (Biognosys, version 13.15.200430.43655) applying the default Biognosys factory settings for DIA analysis. To get a deeper proteome a hybrid spectral library strategy^71^ was followed using the DIA measurements as a project-specific library harboring 55,697 precursors (4,108 protein groups) and an ESC/EpiLC-specific Data-dependent acquisition (DDA) library with in total 230,581 precursors and 9,158 protein groups.

## Supplementary information

Supplementary Information 1.

Supplementary Information 2.

Supplementary Information 3.

## Data Availability

Full proteome data generated in this study can be found in Supplementary Table S2. RNA-seq data generated in this study are available under the accession number E-MTAB-6797 at ArrayExpress https://www.ebi.ac.uk/arrayexpress/ and Supplementary Table S3.
