# Supplementary material for: Distinct and stage-specific contributions of TET1 and TET2 to stepwise cytosine oxidation in the transition from naive to primed pluripotency
Source: Sci Rep. 2020 Jul 21;10:12066. doi: 10.1038/s41598-020-68600-3 (PMC7374584; doi:10.1038/s41598-020-68600-3)
Supplement: Supplementary file 1 — Supplementary Information 1. [file 41598_2020_68600_MOESM1_ESM.pdf]

Supplementary Material

Distinct and stage-specific contributions of TET1 and TET2 to stepwise cytosine oxidation in the transition from naive to primed pluripotency

Christopher B. Mulholland[1], Franziska R. Traube[2], Enes Ugur[2], Edris Parsa[2], Eva-Maria Eckl[1], Maximilian Schönung[1], Miha Modic[3], Michael D. Bartoschek[1], Paul Stolz[1], Joel Ryan[1], Thomas Carell[2], Heinrich Leonhardt*[1] and Sebastian Bultmann*[1]

[1]Department of Biology II and Center for Integrated Protein Science Munich (CIPSM), Ludwig-Maximilians-Universität München, Planegg-Martinsried, Germany

[2]Center for Integrated Protein Science (CIPSM) at the Department of Chemistry, Ludwig-Maximilians-Universität München, Munich, Germany

[3]The Francis Crick Institute, London NW1 1AT, United Kingdom

* correspondence can be addressed to Sebastian Bultmann (bultmann@bio.lmu.de) and Heinrich Leonhardt (h.leonhardt@lmu.de)

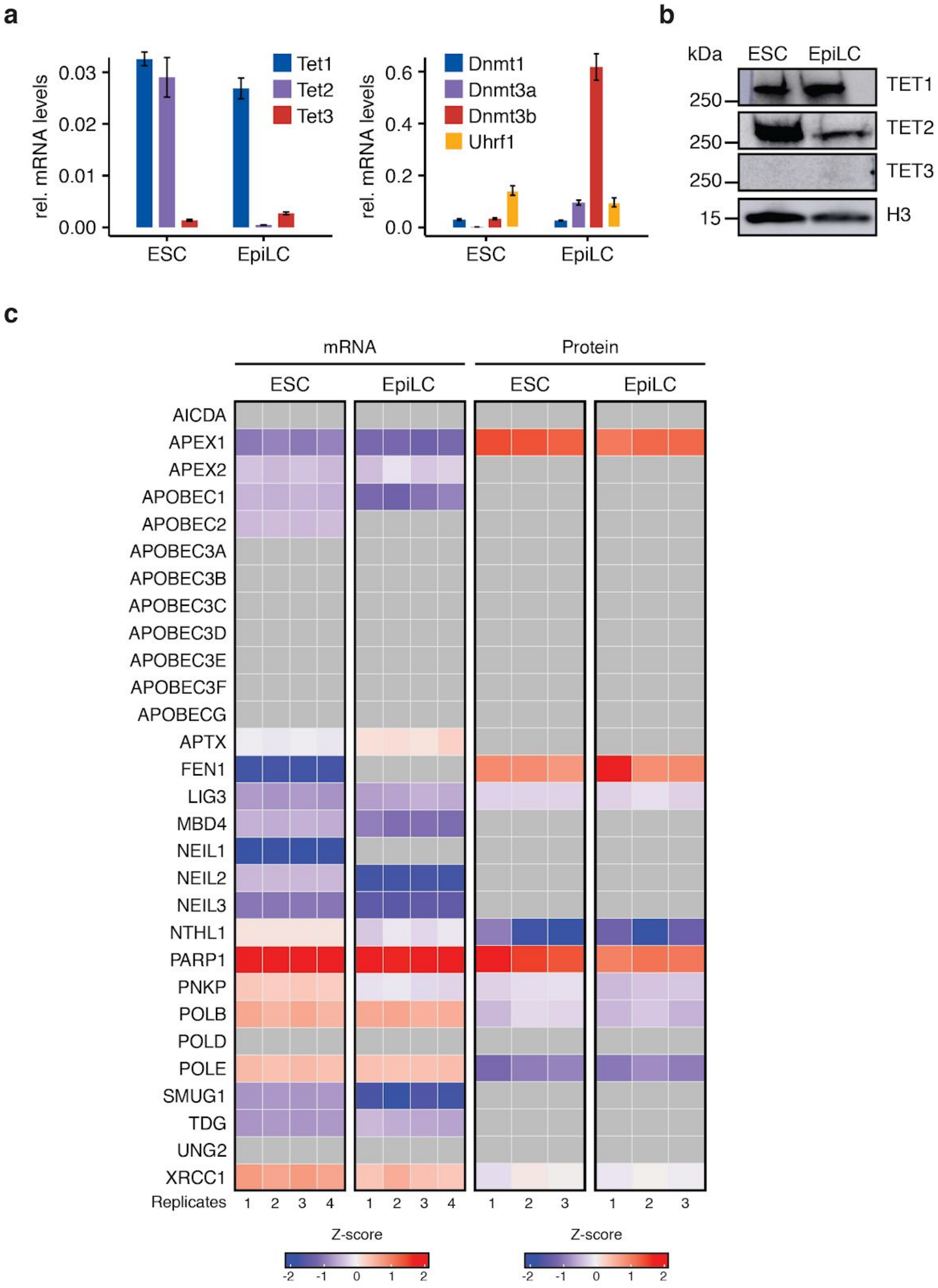
a
b
c
rel. mRNA levels
0.03
0.02
0.01
0.00
Tet1
Tet2
Tet3
ESC
EpiLC
0.6
0.4
0.2
0.0
Dnmt1
Dnmt3a
Dnmt3b
Uhrf1
kDa
250
15
TET1
TET2
TET3
H3
mRNA
Protein
AICDA
APEX1
APEX2
APOBEC1
APOBEC2
APOBEC3A
APOBEC3B
APOBEC3C
APOBEC3D
APOBEC3E
APOBEC3F
APOBECG
APTX
FEN1
LIG3
MBD4
NEIL1
NEIL2
NEIL3
NTHL1
PARP1
PNKP
POLB
POLD
POLE
SMUG1
TDG
UNG2
XRCC1
Replicates
1 2 3 4
1 2 3
Z-score
-2 -1 0 1 2

Supplementary Figure S1: DNA modification dynamics during the naive to primed transition are accompanied by changes in the expression of DNA modifying enzymes.

(a) Expression of DNA modifying enzymes in mESCs and mEpiLCs shown as the relative mRNA levels as a proportion of *Gapdh* at each stage of pluripotency. Error bars indicate mean ± SD calculated from technical triplicate reactions from n = 3 biological replicates.

(b) Western blot analysis of TET1, TET2, TET3 protein levels in wild-type ESCs and EpiLCs with histone H3 as loading control. Immunoblots were repeated 3 times with similar results obtained.

(c) Heatmaps depicting the mRNA levels (left) and protein abundance (right) of DNA repair factors in wild-type ESCs and EpiLCs. Z-scored (Z-score) transcript and protein levels are shown for individual biological replicates (*n* indicated at the bottom of the plots). Gray boxes are used for transcripts and proteins not detected in individual samples by RNA-seq or proteomics measurements, respectively.

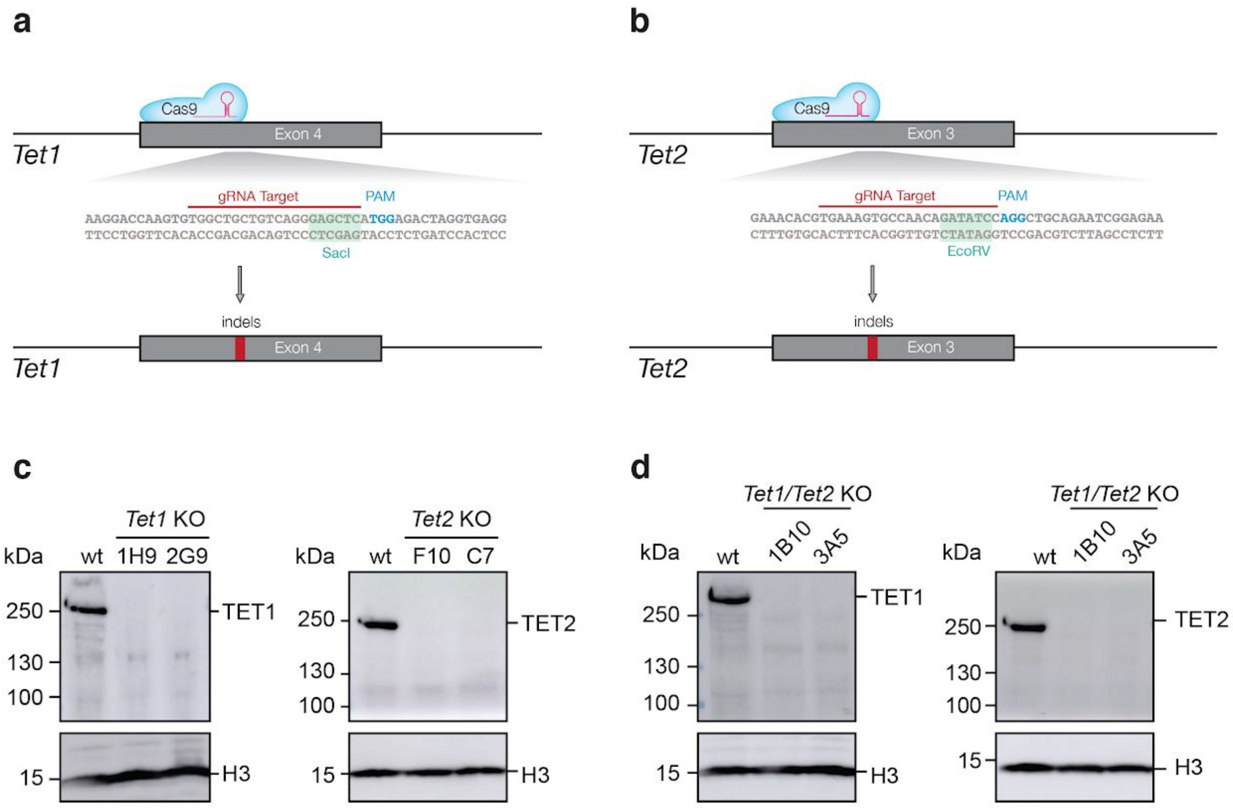

Supplementary Figure S2: Generation and characterization of *Tet* KO cell lines

(a-b) Schematic representation of CRISPR/Cas9 targeting of *Tet1* (**a**) and *Tet2* (**b**) loci for KO generation using gRNA target sequences from [61]. For each gRNA, the PAM (NGG) and specific target sequence are indicated, as well as the location of the restriction enzyme recognition sites used for restriction fragment length polymorphism (RFLP) screening.

(**c-d**) Western blot analysis of TET1 and TET2 protein levels in *Tet1* KO and *Tet2* KO (**c**) and *Tet1*/*Tet2* DKO (**d**) cell lines with histone H3 as loading control. For each *Tet* KO, two independent clones were validated and used in all subsequent experiments. The clones validated via Western blot are as follows: *Tet1* KO (1H9 and 2G9), *Tet2* KO (F10 and C7), and *Tet1*/*Tet2* DKO (1B10 and 3A5). Immunoblots were repeated 3 times with similar results obtained.

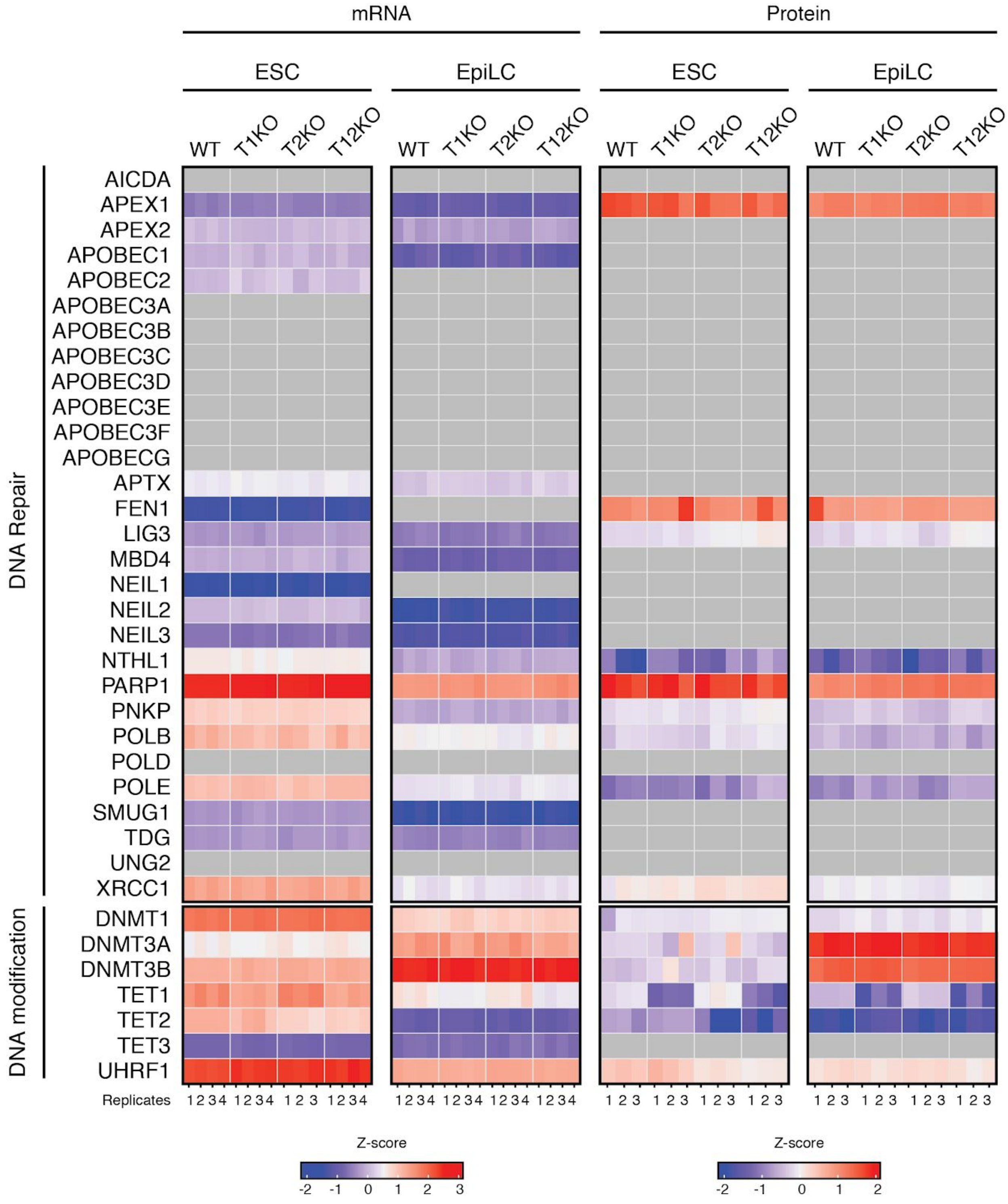
mRNA
Protein
ESC
EpiLC
ESC
EpiLC
WT
T1KO
T2KO
T12KO
DNA Repair
DNA modification
AICDA
APEX1
APEX2
APOBEC1
APOBEC2
APOBEC3A
APOBEC3B
APOBEC3C
APOBEC3D
APOBEC3E
APOBEC3F
APOBECG
APTX
FEN1
LIG3
MBD4
NEIL1
NEIL2
NEIL3
NTHL1
PARP1
PNKP
POLB
POLD
POLE
SMUG1
TDG
UNG2
XRCC1
DNMT1
DNMT3A
DNMT3B
TET1
TET2
TET3
UHRF1
Replicates
1 2 3 4
1 2 3
Z-score
-2 -1 0 1 2 3
Z-score
-2 -1 0 1 2

Supplementary Figure S3: Comparison of mRNA and protein levels for DNA repair factors and DNA modifying enzymes among Tet KO ESCs and EpiLCs

Heatmaps depicting the mRNA levels (left) and protein abundance (right) of DNA repair and modification factors in wild-type (WT), *Tet1 KO* (T1KO), *Tet2 KO* (T2KO), and *Tet1*/*Tet2* DKO (T12KO) ESCs and EpiLCs. Z-scored (Z-score) transcript and protein levels are shown for individual biological replicates (*n* indicated at the bottom of the plots). Gray boxes are used for transcripts and proteins not detected in individual samples by RNA-seq or proteomics measurements, respectively.

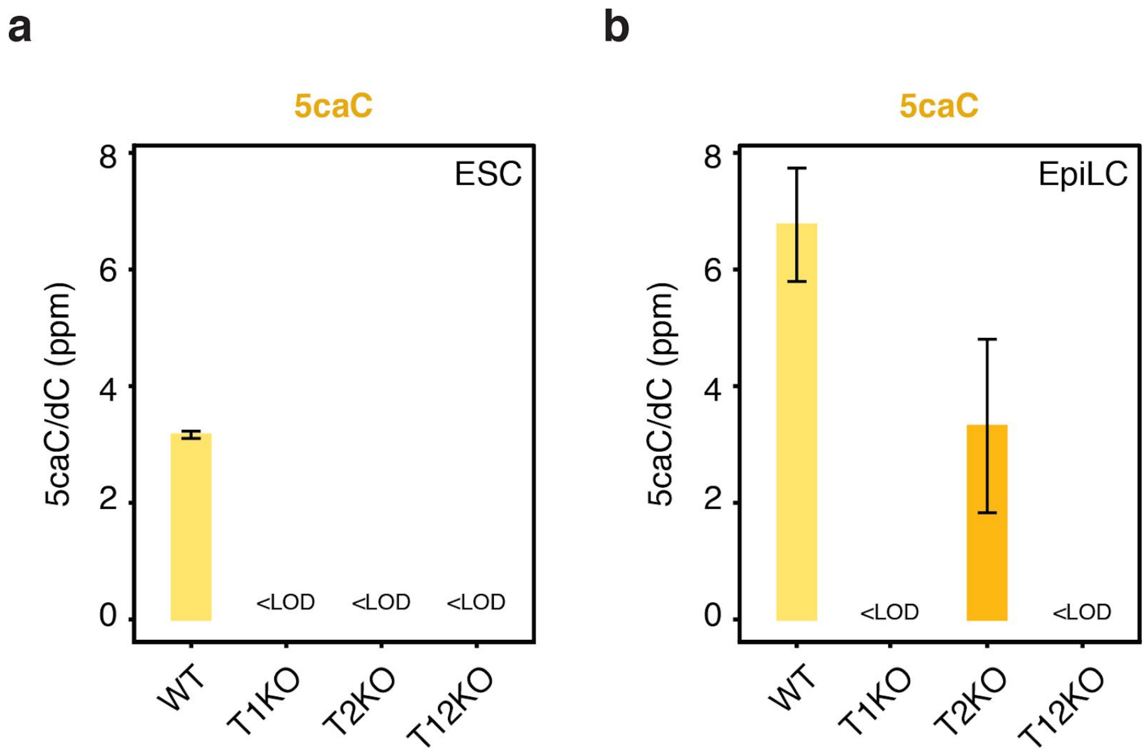

Supplementary Figure S4: Global 5caC levels in *Tet* KO ESCs and EpiLCs

(a,b) Global levels of 5caC in wild-type (WT), *Tet1* KO (T1KO), *Tet2* KO (T2KO), and *Tet1*/*Tet2* DKO (T12KO) ESCs **(a)** and EpiLCs **(b)** as determined by mass spectrometry (UHPLC-MS/MS). 5caC levels are expressed as parts per million (ppm: 1 ppm = 0.0001%) of total cytosine (dC). Error bars indicate mean ± SD calculated from n = 6 biological replicates for each genotype. LOD, limit of detection.

Supplementary Figure S5

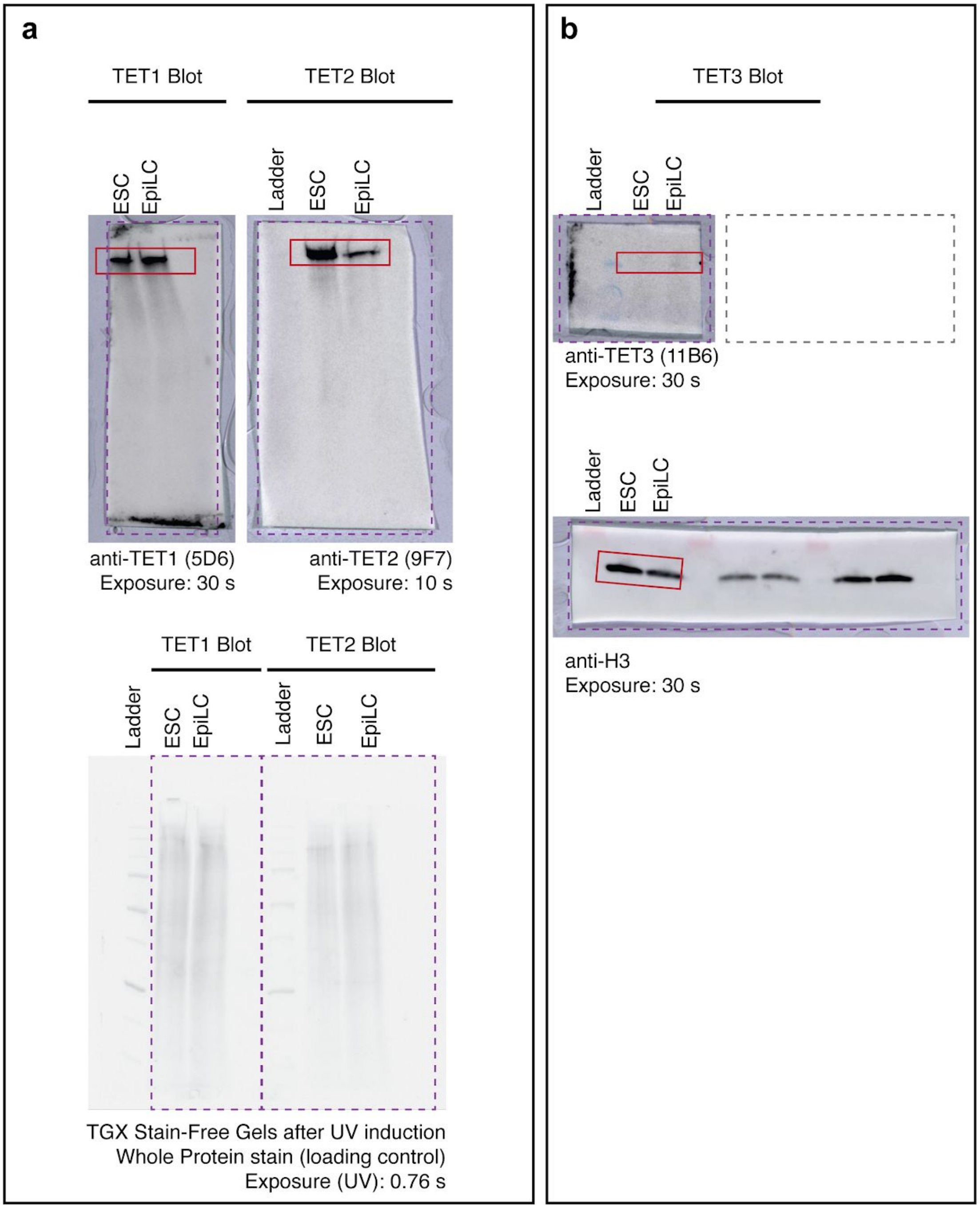

Supplementary Figure S5: Original, uncropped Western Blots from Supplementary Fig. S1b

Original, uncropped Western blots of TET1, TET2, and TET3 protein levels in wild-type ESCs and EpiLCs displayed in Supplementary Fig. S1b with whole protein stain (Tet1 and Tet2) or histone H3 (Tet3) serving as loading controls. Cropped areas are indicated by red boxes. Dotted purple lines delineate the relationship of cut blots. Grey dotted line indicates part of blot removed containing unrelated samples.

Supplementary Figure S6

a **Overlay**

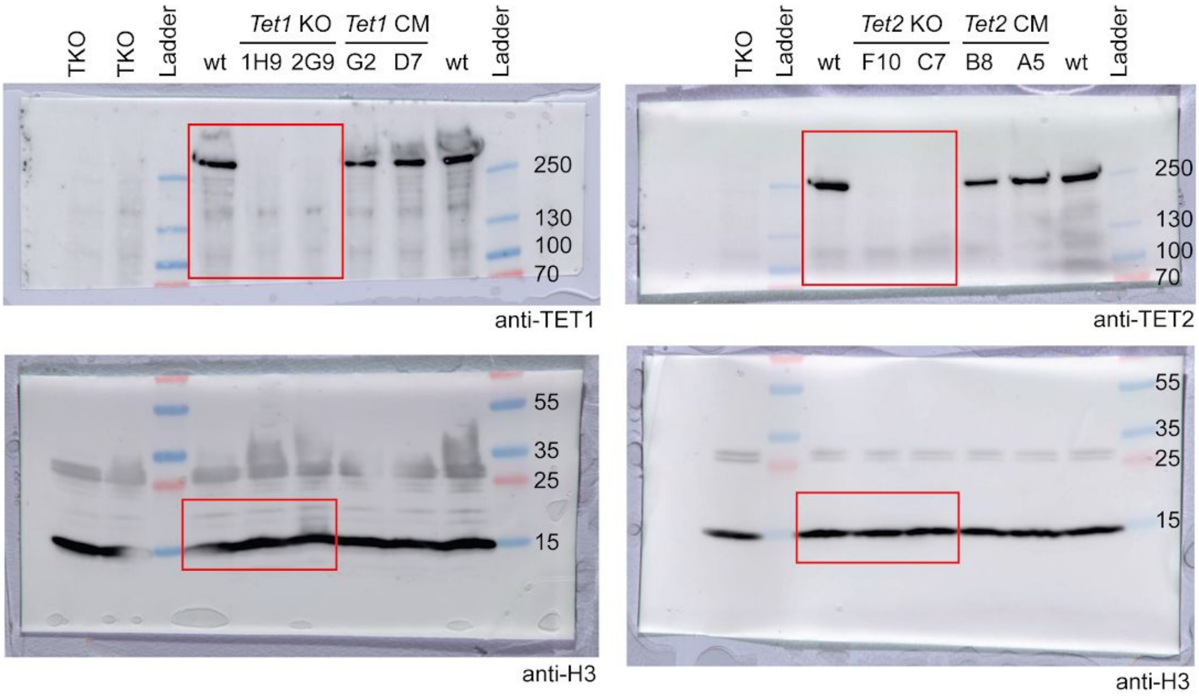

b **Unprocessed**

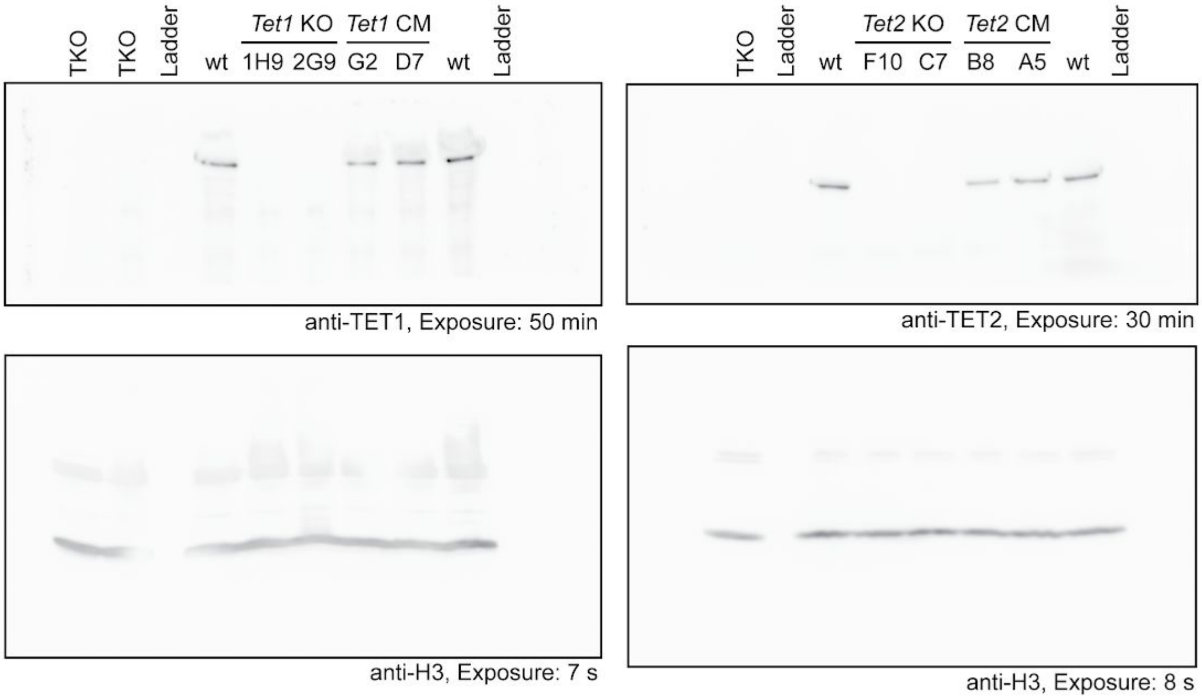

Supplementary Figure S6: Original, uncropped Western Blots from Supplementary Fig. S2c

Overlay (**a**) and unprocessed Western blots with indicated exposure times (**b**) of *Tet1* or *Tet2* single knockout (KO) ESCs displayed in Supplementary Figure S2c with histone H3 as loading control. Cropped areas are indicated by red boxes. Lysates from *Tet1*/*Tet2*/*Tet3* triple knockout (TKO) ESCs [23] are loaded as negative controls. Lysates from wild-type (wt) and *Tet1* or *Tet2* single catalytic mutant (CM) ESCs (unpublished) are loaded as positive controls.

Supplementary Figure S7

a **Overlay**

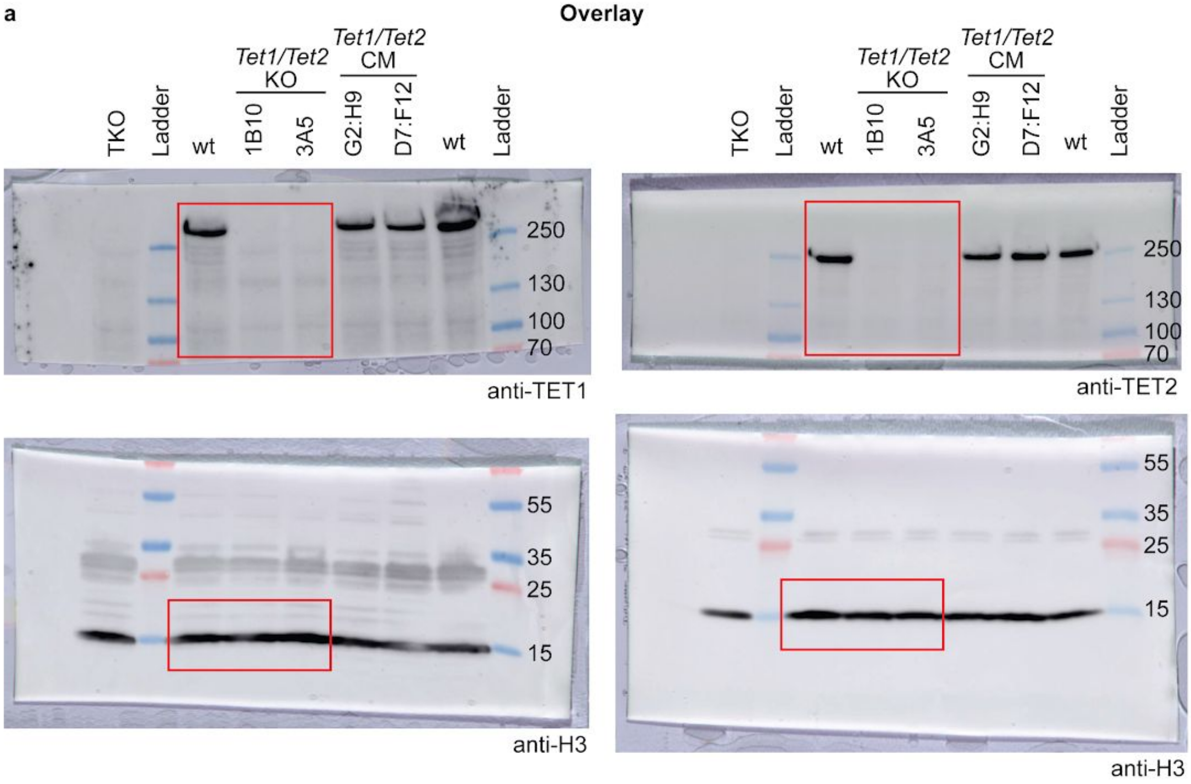

b **Unprocessed**

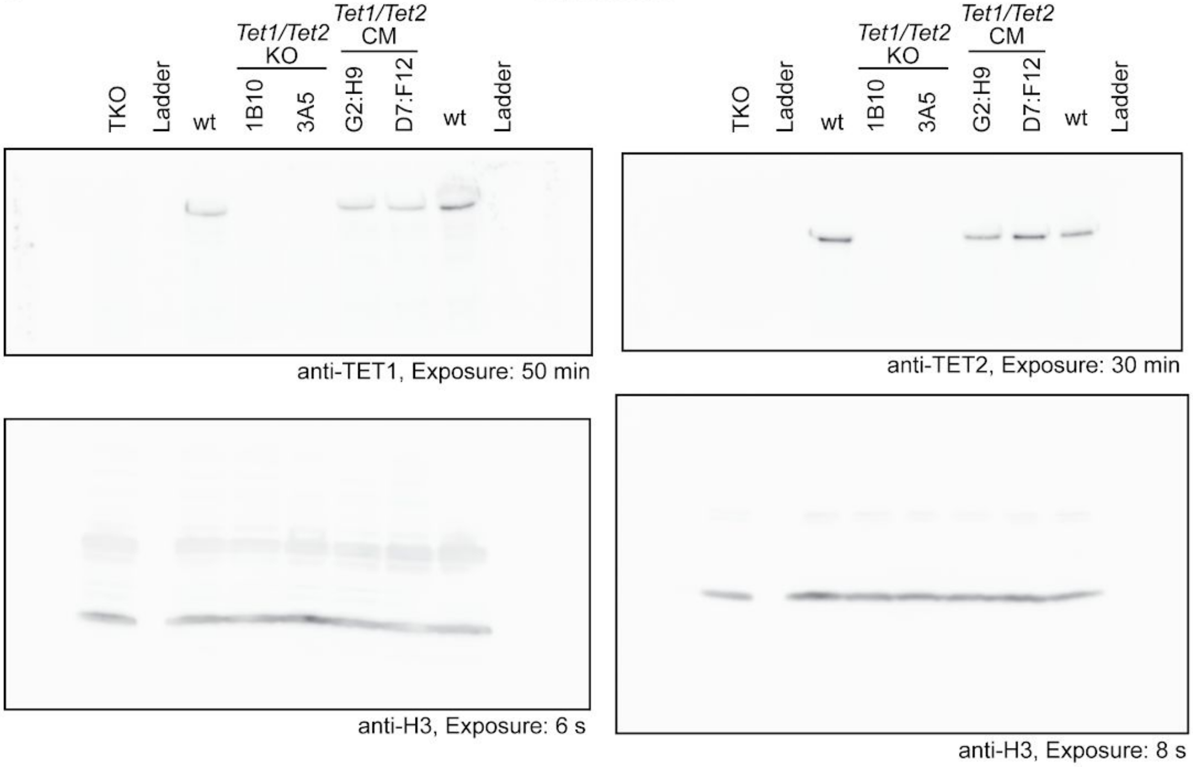

Supplementary Figure S7: Uncropped Western Blots from Supplementary Fig. S2d

Overlay (**a**) and unprocessed Western blots with indicated exposure times (**b**) of *Tet1*/*Tet2* double knockout (KO) ESCs displayed in Supplementary Figure S2d with histone H3 as loading control. Cropped areas are indicated by red boxes. Lysates from *Tet1*/*Tet2*/*Tet3* triple knockout (TKO) ESCs [23] are loaded as a negative control. Lysates from wild-type (wt) and *Tet1*/*Tet2* double catalytic mutant (CM) ESCs (unpublished) are loaded as positive controls.

Supplementary Table S1: Modified Cytosine Level Quantification

condition	genotype	n	5mC/dC (%)	5mC sd	hmC/dC (%)	5hmC sd	fC/dC (ppm)	5fC sd	n	5caC/dC (ppm)	5caC sd
ESC	wt	18	3.1778	0.11707	0.1084	0.00022	5.7778	0.018	6	3.1700	0.0615
ESC	T1KO	18	3.6346	0.08240	0.0533	0.00096	2.8722	0.043	6	<L.O.D	<L.O.D
ESC	T2KO	12	3.9500	0.09779	0.0294	0.00699	0.7250	0.088	6	<L.O.D	<L.O.D
ESC	T12KO	12	3.6250	0.13198	0.0031	0.02495	0.4275	1.467	6	<L.O.D	<L.O.D
EpiLC	wt	24	6.8375	0.13878	0.5148	8.3E-05	17.0000	0.015	6	6.7700	0.9740
EpiLC	T1KO	12	7.7614	0.07211	0.0525	0.00169	2.6480	0.055	6	<L.O.D	<L.O.D
EpiLC	T2KO	12	8.2250	0.04174	0.3087	0.00042	4.4500	0.025	6	3.3200	1.4900
EpiLC	T12KO	12	7.8417	0.04468	0.0073	0.00549	0.3308	0.311	6	<L.O.D	<L.O.D

Supplementary Table S2: Proteome profiling of Tet KO ESCs and EpiLCs
(see spreadsheet)

Supplementary Table S3: Transcriptome profiling of Tet KO ESCs and EpiLCs
(see spreadsheet)

Supplementary Table S4: Modified Cytosine Level Analysis

condition	genotype	5mC [% wt 5mC]	5hmC [% wt 5hmC]	5fC [% wt 5fC]
ESC	T1KO	114.4 ± 2.6	49.1 ± 0.9	49.7 ± 0.7
ESC	T2KO	124.3 ± 3.1	27.1 ± 6.4	12.5 ± 1.5
ESC	T12KO	114.1 ± 4.2	2.8 ± 23.0	7.4 ± 2.4
EpiLC	T1KO	113.5 ± 1.1	10.2 ± 0.3	15.6 ± 0.3
EpiLC	T2KO	120.3 ± 0.6	60.0 ± 0.1	26.2 ± 0.1
EpiLC	T12KO	114.7 ± 0.7	1.4 ± 1.1	1.9 ± 1.8

Supplementary Table S5: Oligonucleotides

**RT-qPCR Primers**	
**Name**	**Sequence**
Tet1_F	CCAGGAAGAGGCGACTACGTT
Tet1_R	TTAGTGTTGTGTGAACCTGATTTATTGT
Tet2_F	ACTTCTCTGCTCATTCCCACAGA
Tet2_R	GGTGCCTCTGGAGTGTTGGT
Tet3_F	GAGCACGCCAGAGAAGATCAA
Tet3_R	CAGGCTTTGCTGGGACAATC
Dnmt1_F	GGCGGAAATCAAAGGAGGAT
Dnmt1_R	CCTGGGTCTGGAACTTCTTTTATC
Dnmt3a_F	AAGTGCAGAAACATCGAGGACAT
Dnmt3a_R	CTGGCACATGCCTCCAATG
Dnmt3b_F	CCCGTTCGACTTGGTGATTG
Dnmt3b_R	CTTCCTGTGCCCTCATATAAACCT
Uhrf1_F	GGCAGCTGAAGCGGATGA
Uhrf1_R	CCATGCACCGAAGATATTGTCA
**Oligonucleotides for gRNA Cloning**	
**Name**	**Sequence**
Tet1_KO_gRNA_F	CACCGGCTGCTGTCAGGGAGCTCA
Tet1_KO_gRNA_R	AAACTGAGCTCCCTGACAGCAGCC
Tet2_KO_gRNA_F	CACCGAAAGTGCCAACAGATATCC
Tet2_KO_gRNA_R	AAACGGATATCTGTTGGCACTTTC
**Oligonucleotides for RFLP Analysis**	
**Name**	**Sequence**
Tet1KO_scrF	TTGTTCTCTCCTCTGACTGC
Tet1KO_scrR	TGATTGATCAAATAGGCCTGC
Tet2KO_scrF	CAGATGCTTAGGCCAATCAAG
Tet2KO_scrR	AGAAGCAACACACATGAAGATG

Supplementary Table S6: Compound-dependent LC-MS/MS-parameters used for the analysis of genomic DNA

compound	Precursor ion (m/z)	MS1 Resolution	Product Ion (m/z)	MS2 Resolution	Dwell time [ms]	CE (V)	CAV (V)	Polarity
**Time segment 1.5-4.2 min**								
[$^{15}N_2$]-cadC	274.08	Wide	158.03	Wide	60	5	5	Positive
cadC	272.09	Wide	156.04	Wide	60	5	5	Positive
[$^{15}N_2$]-caC	158.03	Wide	140.09	Wide	60	13	7	Positive
caC	156.04	Wide	138.03	Wide	60	13	7	Positive
[$^{15}N_2$,D_2]-hmdC	262.12	Wide	146.07	Wide	60	27	1	Positive
hmdC	258.11	Wide	142.06	Wide	60	27	1	Positive
[D_3]-mdC	245.13	Wide	129.09	Wide	60	60	1	Positive
mdC	242.11	Wide	126.07	Wide	60	60	1	Positive
dC	228.10	Wide	112.05	Wide	1	1	0	Positive
**Time segment 4.2-9 min**								
[$^{15}N_5$]-8-oxo-dG	289.08	Wide	173.04	Wide	80	9	7	Positive
8-oxo-dG	284.10	Wide	168.05	Wide	80	9	7	Positive
[$^{15}N_2$]-fdC	258.09	Wide	142.04	Wide	80	5	5	Positive
fdC	256.09	Wide	140.05	Wide	80	5	5	Positive
[$^{15}N_2$]-fC	142.04	Wide	98.04	Wide	80	13	7	Positive
fC	140.05	Wide	97.04	Wide	80	13	7	Positive

CE: collision energy, CAV: collision cell accelerator voltage, EMV: electron multiplier voltage. The nucleosides were analyzed in the positive ($[M+H)]^+$ species) as well as the negative ($[M-H]^-$ species) ion selected reaction monitoring mode (SRM).
